# Assessment of Neonatal Intensive Care Unit Practices and Preterm Newborn Gut Microbiota and 2-Year Neurodevelopmental Outcomes

**DOI:** 10.1001/jamanetworkopen.2020.18119

**Published:** 2020-09-23

**Authors:** Jean-Christophe Rozé, Pierre-Yves Ancel, Laetitia Marchand-Martin, Clotilde Rousseau, Emmanuel Montassier, Céline Monot, Karine Le Roux, Marine Butin, Matthieu Resche-Rigon, Julio Aires, Josef Neu, Patricia Lepage, Marie-José Butel

**Affiliations:** 1 Neonatal Department, INSERM-CHU Clinical Investigation Center 1413, et UMR- INRA 1280, Physiologie des Adaptations Nutritionnelles, Nantes University Hospital, Nantes, France; 2 Université de Paris, Center for Epidemiology and Statistics/CRESS U1153/EPOPé Team, Paris, France; 3 Clinical Investigation Center P1419, Assistance Publique-Hôpitaux de Paris, Paris, France; 4 UMR-S INSERM U1139, Faculté de Pharmacie, Université de Paris, Paris, France; 5 PremUp Foundation, Paris, France; 6 Microbiology Department, AP-HP Hôpital Saint-Louis, Paris, France; 7 Service des Urgences, Nantes University Hospital, Nantes, France; 8 Micalis Institute, INRA, AgroParisTech, University Paris-Saclay, Paris, France; 9 Neonatal Department, Hospices Civils de Lyon, Lyon, France; 10 Biostatistics and Medical Information Department, AP-HP Hôpital Saint-Louis, Paris, France; 11 College of Medicine, University of Florida, Gainesville, Florida

## Abstract

**Importance:**

In very preterm newborns, gut microbiota is highly variable with major dysbiosis. Its association with short-term health is widely studied, but the association with long-term outcomes remains unknown.

**Objective:**

To investigate in preterm newborns the associations among practice strategies in neonatal intensive care units (NICUs), gut microbiota, and outcomes at 2 years.

**Design, Setting, and Participants:**

EPIFLORE is a prospective observational cohort study that includes a stool sample collection during the fourth week after birth. Preterm newborns of less than 32 weeks of gestational age (GA) born in 2011 were included from 24 NICUs as part of the French nationwide population-based cohort, EPIPAGE 2. Data were collected from May 2011 to December 2011 and analyzed from September 2016 to December 2018.

**Exposures:**

Eight NICU strategies concerning sedation, ventilation, skin-to-skin practice, antibiotherapy, ductus arteriosus, and breastfeeding were assessed. A NICU was considered favorable to a practice if the percentage of that practice in the NICU was more than the expected percentage.

**Main Outcomes and Measures:**

Gut microbiota was analyzed by 16S ribosomal RNA gene sequencing and characterized by a clustering-based method. The 2-year outcome was defined by death or neurodevelopmental delay using a Global Ages and Stages questionnaire score.

**Results:**

Of 577 newborns included in the study, the mean (SD) GA was 28.3 (2.0) weeks, and 303 (52.5%) were male. Collected gut microbiota was grouped into 5 discrete clusters. A sixth cluster included nonamplifiable samples owing to low bacterial load. Cluster 4 (driven by *Enterococcus* [n = 63]), cluster 5 (driven by *Staphylococcus* [n = 52]), and cluster 6 (n = 93) were significantly associated with lower mean (SD) GA (26.7 [1.8] weeks and 26.8 [1.9] weeks, respectively) and cluster 3 (driven by *Escherichia/Shigella* [n = 61]) with higher mean (SD) GA (29.4 [1.6] weeks; *P* = .001). Cluster 3 was considered the reference. After adjustment for confounders, no assisted ventilation at day 1 was associated with a decreased risk of belonging to cluster 5 or cluster 6 (adjusted odds ratio [AOR], 0.21 [95% CI, 0.06-0.78] and 0.19 [95% CI, 0.06-0.62], respectively) when sedation (AOR, 10.55 [95% CI, 2.28-48.87] and 4.62 [1.32-16.18], respectively) and low volume of enteral nutrition (AOR, 10.48 [95% CI, 2.48-44.29] and 7.28 [95% CI, 2.03-26.18], respectively) was associated with an increased risk. Skin-to-skin practice was associated with a decreased risk of being in cluster 5 (AOR, 0.14 [95% CI, 0.04-0.48]). Moreover, clusters 4, 5, 6 were significantly associated with 2-year nonoptimal outcome (AOR, 6.17 [95% CI, 1.46-26.0]; AOR, 4.53 [95% CI, 1.02-20.1]; and AOR, 5.42 [95% CI, 1.36-21.6], respectively).

**Conclusions and Relevance:**

Gut microbiota of very preterm newborns at week 4 is associated with NICU practices and 2-year outcomes. Microbiota could be a noninvasive biomarker of immaturity.

## Key Points

**Question:**

What are the long-term outcomes associated with dysbiosis of gut microbiota in very preterm newborns?

**Findings:**

In this cohort study of 577 very preterm newborns across 24 neonatal intensive care units from a French nationwide cohort, gut microbiota at week 4 after birth showed 6 bacterial patterns that varied according to gestational age, perinatal characteristics, individual treatments, and neonatal intensive care unit strategies. Three clusters were associated with 2-year outcomes after adjustment for these confounders.

**Meaning:**

Modifying strategies associated with alterations in microbiota, such as promoting enteral nutrition, reducing sedation use, promoting early extubation, or skin-to-skin practice, may be correlated with outcomes in preterm newborns.

## Teaser

This cohort study of 577 very preterm newborns across 24 neonatal intensive care units from a French nationwide cohort investigates associations among practice strategies, gut microbiota, and outcomes at 2 years.

## Introduction

Knowledge about the role in host health of the gut intestinal microbiota has considerably improved with advances in culture-independent and sequencing technologies.^1^ Hence, microbiome analysis could provide new markers of disease that would make great advances in patient care.^2^ The microbiota has also been proposed as a major feature in critically ill adult patients and constitutes a key therapeutic target for the prevention and treatment of critical illness.^3^

The dynamic bacterial establishment from birth to around 3 years of age is influenced by several perinatal determinants and may lead to lifelong signature with potential effects on health.^1,4^ In preterm newborns, several studies have shown that gut microbiota is different from that of term newborns, with high variable colonization patterns. Gestational age at birth still imprints on the microbiome up to 4 years of age.^5^ Gut microbiota in this at-risk population has mainly been investigated in studies focusing on meconium,^4^ on factors effecting the bacterial establishment,^6,7,8,9,10,11^ or on dysbiosis prior to or at the onset of necrotizing enterocolitis.^12,13^ However, while influence on the bacterial establishment of the neonatal intensive care unit (NICU) has been reported,^10,14,15,16^ very few data are available on the relationship between neonatal microbiota and practices, and on the potential effect on further outcome in extremely preterm newborns.^17,18^

EPIPAGE 2,^19^ a nationwide prospective population-based cohort study that included very preterm newborns, procured a unique opportunity to assess variation of practice^20,21^ among NICUs and to perform an ancillary study, EPIFLORE, on stool samples collected during the fourth week after birth of preterm newborns in voluntary NICUs. We hypothesized that microbiota varies according to the practices and is associated with further outcome.

## Methods

### Cohort EPIPAGE 2 and Ancillary EPIFLORE Studies

EPIPAGE 2 was performed in 68 NICUs in France and included newborns born at 24 to 31 weeks of gestation. The EPIFLORE study is an ancillary study of EPIPAGE 2 and consists of the establishment of a collection of stools carried out in a subset of 24 voluntary NICUs. Eligible children for the current study were those alive at week 4 after birth and hospitalized in these 24 NICUs. The results from this study were analyzed and reported in accordance with the Strengthening the Reporting of Observational Studies in Epidemiology (STROBE) reporting guideline.^22^

### Ethics

Recruitment and data collection occurred only after families had received information and agreed to participate in this cohort by oral informed consent as recommended by French law in case of noninterventional research. The study was approved by the National Data Protection Authority, by the Consultative Committee on the Treatment of Information on Personal Health Data for Research Purposes, and by the Committee for the Protection of People Participating in Biomedical Research.

### Perinatal and Neonatal Characteristics

In each center, 1 obstetric and 1 pediatric study coordinator were responsible for data acquisition, validation, and quality control. Data were collected prospectively during hospitalization until discharge. Extensive data were collected about pregnancy, delivery, and the neonatal period.

### Strategies of NICUs

Eight NICUs’ strategies concerning early extubation or no intubation, use of sedation, direct breastfeeding, skin-to-skin practice, treatment of ductus arteriosus, speed of progression of enteral feeding, and duration of primary and secondary antibiotherapy were characterized in EPIPAGE 2. All of these practices concern the early period of hospitalization, before stool samples were obtained. For each infant and for each strategy, a probability to receive it was calculated by logistic regression according to the characteristics of the preterm newborn and their mother concerned by this strategy (see eMethods in the Supplement) as previously described.^20^ From the average of the probabilities of receiving treatment for children from the same NICU, we calculated an expected percentage to have this strategy applied in this NICU. If the observed difference in percentage was zero or greater than the expected percentage, the NICU strategy was considered as favorable to the application of this strategy. If the difference was negative, the strategy was considered unfavorable.

### 2-Year Outcome

The 2-year outcome investigated was death after week 4 of life or the newborn’s neurodevelopment at 2 years of age. Data for children at 2 years of age (corrected age for prematurity) were collected by using 2 standardized questionnaires^23^: 1 survey completed by the referring physician to assess cerebral palsy and, the other completed by the parents to assess overall neurodevelopment using the second version of the 24-month Ages and Stages questionnaire (ASQ) already validated in France.^24^ A nonoptimal 2-year outcome was defined by death or a development delay based on ASQ score of less than 185 at 2 years,^25^ or by death or cerebral palsy in secondary analyses.^23^ Death and 2-year outcome are united in a single outcome because of their competitive nature.

### Microbiological Analysis of Fecal Samples

Fresh fecal samples were collected from diapers during week 4 after birth and immediately stored at −80 °C until microbiota analysis. This time of sampling had been chosen to take into account a sufficient time of exposure to a NICU’s practices. Microbiota composition was analyzed using 16S ribosomal RNA gene sequencing with MiSeq (Illumina). Total DNA was extracted according to International Human Microbiome Standards standard operating procedure 7.^26^ For amplicons sequencing, we used the V3 and V4 primers (V3fwd: TACGGRAGGCAGCAG, V4rev: TACCAGGGTATCTAAT;).^27^ Positive and negative polymerase chain reaction controls were added to each sequencing libraries. The raw sequences were analyzed using the open source software package Quantitative Insights Into Microbial Ecology.^28^ After trimming primers and barcodes, the sequences were filtered for quality (minimum length = 200 bp, minimum quality threshold = 20, chimeras removal) and clustered into operational taxonomic units (OTUs) at a threshold of 97% similarity level using uclust. The OTUs represented by fewer than 3 reads were removed from the OTU table. Samples amplified but resulting in fewer than 1000 reads were also removed (n = 3). The most abundant member of each OTU was selected as the representative sequence and assigned to different taxonomic levels using the Ribosomal Database Project naive bayesian classifier and Ribosomal Database Project Seqmatch program.^29^

### Statistical Analysis

First, to ensure the representativeness of the population sample studied, we compared the population of preterm newborns enrolled with the nonenrolled eligible population from the EPIFLORE study (ie, newborns hospitalized in the 24 NICUs participating in the EPIFLORE study but without stool collection). Second, to describe microbiota, newborns’ gut microbiota was stratified by clustering methods based on taxonomic composition at the genus level. Relative abundance profiles were clustered by the partitioning around medoids algorithm.^27^ Optimal clusters number was assessed by the Calinski-Harabasz score. A supplemental cluster (cluster 6) was defined by newborns with lack of DNA amplification owing to low bacterial load.

Third, in order to analyze the association between microbiota clusters and perinatal, neonatal characteristics and the exposition to the 8 studied strategies in NICUs where newborns were hospitalized at day 7, we performed univariable and multivariable analyses, using multinomial mixed-effects logistic regression with a random hospital intercept to take into account the correlation between newborns of the same NICU.

Fourth, in order to analyze the association between microbiota cluster and the 2-year outcome, we performed 3 mixed-effects logistic regressions: (1) with adjustment for gestational age (GA); (2) for GA, characteristics of the mother, perinatal characteristics of the newborn, and received treatments; and (3) for GA, characteristics of the mother, perinatal characteristics of the newborn, and the 8 strategies of the NICU where the newborn was hospitalized at day 7. Management of missing data was based on multiple imputations. Missing data were imputed performing fully conditional specification method with SAS software, version 9.4 (SAS Institute) MI procedure. Imputation model variables included baseline mother and newborn characteristics, individual therapeutics received, the NICU’s strategies, gut microbiota clusters, survival outcomes, and 2-year outcomes (assessed by measure of ASQ score). Binary and categorical variables were imputed using logistic regression or multinomial models. The ASQ score was imputed using predictive mean matching. We generated 50 independent imputed data sets with 30 iterations each. Estimates were pooled according to the Rubin rule.

For descriptive analyses, we used weighted percentages to take into account the differences in the recruitment times for the newborns born at 24 to 26 weeks of gestation or at 27 and 31 weeks of gestation in EPIPAGE 2, and not to overrepresent 24 to -26 weeks. Moreover, we performed some sensitivity analyses (ie, analysis without imputation and analysis using GA as continuous variable). All tests were 2-sided, and *P* values less than .05 were considered significant. All analyses were performed with the SAS software (SAS Institute Inc) and R software, version 3.4.3 (R Foundation).

## Results

Fecal samples were collected for 577 preterm newborns at a median age of 23 days (interquartile range [IQR], 22-26 days). These newborns were not significantly different from eligible patients who could not be included in the EPIFLORE project (n = 529; eFigure 1 in the Supplement), except for their GA, the rate of mothers born outside of France, and the proportion of newborns with an irregular transit during the first week (eTable 1 in the Supplement).

Among the 60 NICUs included in EPIPAGE 2 where more than 10 children were hospitalized, we have pointed out differences between NICUs participating and not participating in the EPIFLORE study (eFigure 2 in the Supplement). Among the 18 NICUs participating in the EPIFLORE study, 13 were considered favorable to sedation during the first week, 5 to low volume of enteral feeding during first week, 10 to skin-to-skin contact with parents during first week, 2 to direct breastfeeding during first week, 8 to early extubation at day 1 or no intubation, 11 to use of ibuprofen during the first 10 days to close ductus arteriosus, 7 to longer duration of first antibiotherapy, and 7 to longer duration of secondary antibiotherapy (eFigure 3 in the Supplement).

**Figure 1.**
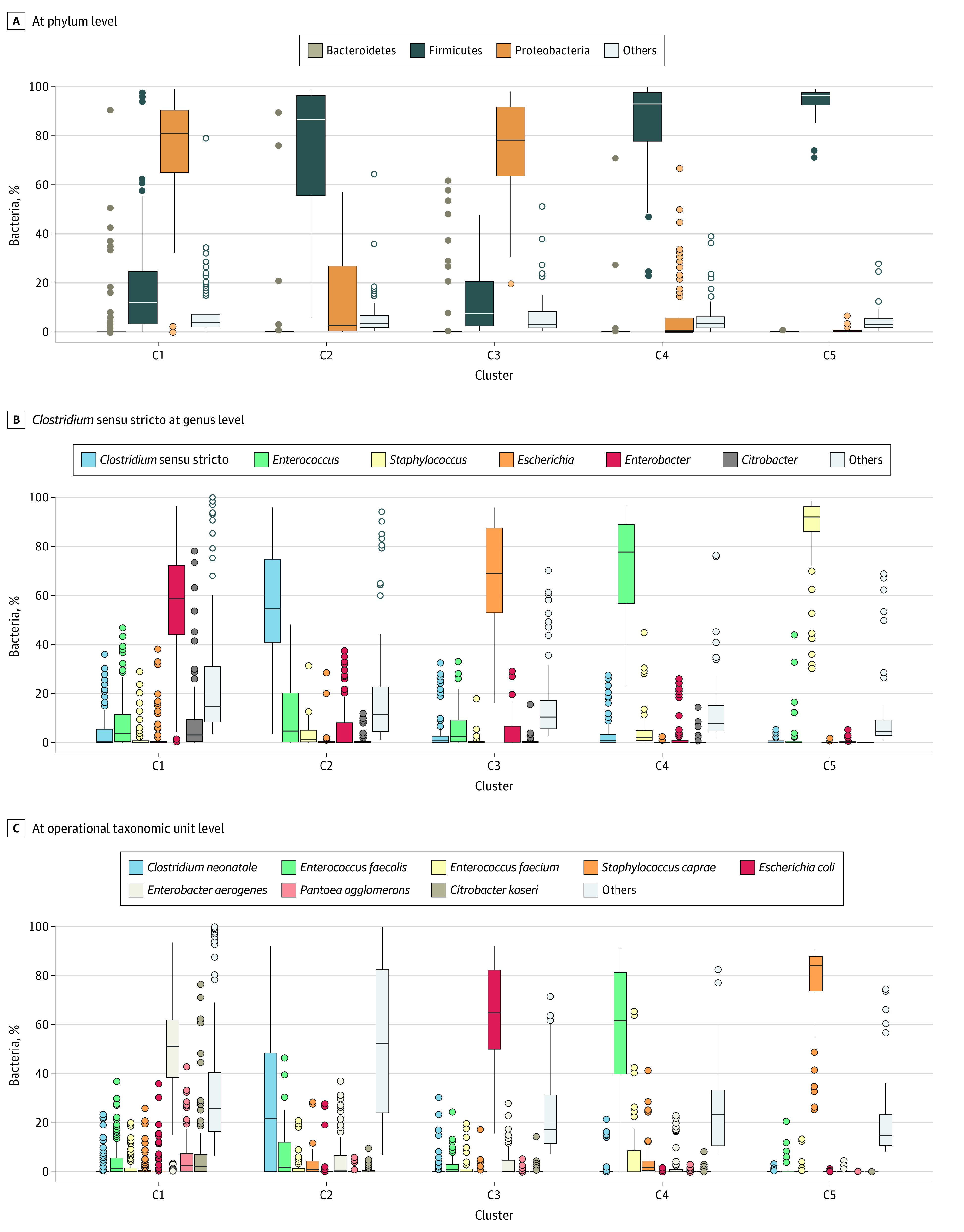
Composition of the 5 Identified Microbiota Clusters Mean composition of each cluster is represented at phylum (A), genus (B), and operational taxonomic unit levels (C). The sixth cluster, constituted by newborns in whom no amplification could be performed owing to a low bacterial load, is not represented by definition.

Among 577 stool samples, microbial DNA could be amplified and analyzed from 484. The median (IQR) Shannon diversity index was low: 2.56 (1.85-3.74). Bacterial patterns were distributed among 5 clusters (Figure 1; eFigure 4 in the Supplement). Bacterial genera driving these clusters were identified by a random forest analysis: dominance of *Enterobacter aerogenes* in cluster 1 (n = 240) with a median (IQR) abundance of 58% (44%-73%), dominance of *Clostridium sensu-stricto* in cluster 2 (n = 68) with a median (IQR) abundance of 55% (40%-77%), dominance of *Escherichia*/*Shigella* in cluster 3 (n = 61) with a median (IQR) abundance of 67% (52%-87%), dominance of *Enterococcus* in cluster 4 (n = 63) with a median (IQR) abundance of 78% (58%-89%), and dominance of *Staphylococcus* in cluster 5 (n = 52) with a median (IQR) abundance of 92% (84%-96%). The 93 newborns with no bacterial DNA amplifications owing to low bacterial load and not associated with a low sample weight or total DNA concentration constituted cluster 6. At the OTU level, a single OTU was dominant in 4 clusters: median abundance (IQR) was respectively 51% (38%-62%) for *E aerogenes*_OTU5990 in cluster 1, 64% (50%-82%) for *Escherichia coli*_OTU7123 in cluster 3, 63% (40%-82%) for *Enterococcus faecalis*_OTU1227 in cluster 4, 84% (73%-86%) for *Staphylococcus caprae*_OTU5825 in cluster 5.

Characteristics of mothers and newborns and individual therapeutics were significantly associated with belonging to 1 cluster in univariate (Table 1) and multivariate (eTable 2 in the Supplement) analysis. Cluster 3, driven by *Escherichia coli*_OTU7123 was associated with higher GA, corresponding therefore to the more mature microbiota, and thus was chosen as the reference cluster. Newborns born to mothers from North Africa were associated with cluster 2. Lower GA was significantly associated with clusters 4, 5, and 6 (Table 1). Birth by cesarean delivery was associated with increased risk of being in clusters 1, 5, and 6. Regular intestinal transit during the first week was associated with reduced risk of being in clusters 4 and 5, and receiving breast milk during the first week was associated with reduced risk of being in clusters 1 or 6. Late-onset infections before stool collection was associated with increased risk of being in cluster 6.

**Table 1.** Associations Between Cluster of Microbiota, Neonatal Characteristics, Individual Therapies, and NICU Strategies

Variable	Cluster, No./No. (%)^a^						*P* value
	1 (n = 240)	2 (n = 68)	3 (n = 61)	4 (n = 63)	5 (n = 52)	6 (n = 93)	
Gestational age, wk							
24-26	33/240 (10.6)	8/68 (9.0)	6/61 (7.5)	20/63 (25.7)	31/52 (52.3)	47/93 (43.1)	<.001
27-29	101/240 (43.6)	25/68 (37.9)	16/61 (26.9)	32/63 (55.3)	17/52 (38.6)	35/93 (43.3)	
30-31	106/240 (45.8)	35/68 (53.1)	39/61 (65.6)	11/63 (19.0)	4/52 (9.1)	11/93 (13.6)	
Maternal age, y							
<25	38/240 (16.0)	6/68 (9.1)	10/61 (16.0)	17/63 (26.7)	10/52 (19.2)	15/93 (16.6)	.03
25-34	136/240 (56.7)	49/68 (71.2)	43/61 (70.6)	35/63 (56.9)	31/52 (59.3)	48/93 (51.1)	
>35	66/240 (27.3)	13/68 (19.7)	8/61 (13.5)	11/63 (16.3)	11/52 (21.5)	30/93 (32.3)	
Country of birth of the mother							
France	174/237 (73.0)	46/68 (67.8)	51/61 (83.6)	48/63 (75.4)	32/52 (63.3)	69/93 (74.5)	.001
North Africa countries	20/237 (8.5)	18/68 (26.5)	3/61 (5.0)	3/63 (4.7)	6/52 (10.7)	9/93 (9.5)	
Other African countries	26/237 (11.1)	1/68 (1.1)	4/61 (6.3)	6/63 (9.9)	9/52 (16.9)	11/93 (11.4)	
Other	17/237 (7.3)	3/68 (4.5)	3/61 (5.0)	6/63 (9.9)	5/52 (9.0)	4/93 (4.6)	
Maternal level of education							
<Higher secondary school	64/222 (28.8)	30/66 (45.7)	15/60 (24.7)	14/58 (24.3)	9/46 (18.6)	19/72 (27.9)	.06
Higher secondary school	39/222 (17.6)	12/66 (17.6)	15/60 (25.5)	13/58 (21.5)	13/46 (27.5)	16/72 (21.5)	
High school diploma +1 +2	53/222 (23.7)	9/66 (14.1)	9/60 (14.9)	20/58 (35.1)	10/46 (23.1)	19/72 (26.3)	
>High school diploma +3	66/222 (29.9)	15/66 (22.7)	21/60 (34.9)	11/58 (19.2)	14/46 (30.8)	18/72 (24.3)	
Maternal BMI before pregnancy							
Underweight	16/222 (7.2)	4/64 (6.5)	4/59 (6.5)	3/60 (5.0)	4/47 (8.7)	7/83 (9.0)	.49
Underweight or normal	138/222 (62.0)	36/64 (56.0)	36/59 (61.3)	38/60 (63.8)	28/47 (59.0)	46/83 (55.0)	
Overweight	40/222 (17.8)	8/64 (12.5)	13/59 (22.2)	14/60 (22.6)	11/47 (24.3)	19/83 (22.8)	
Obese	28/222 (13.0)	16/64 (25.0)	6/59 (10.0)	5/60 (8.6)	4/47 (8.1)	11/83 (13.1)	
Neonatal factors							
Male	121/240 (50.3)	32/68 (46.2)	35/61 (57.6)	37/63 (60.0)	33/52 (64.4)	42/93 (45.2)	.15
Birth weight Z-score, mean (SD)^b^	240 0.1 (1.0)	68 0.4 (1.1)	61 0.1 (1.0)	63 0.4 (0.9)	52 0.4 (1.1)	93 0.3 (1.1)	.03
Cesarean delivery	166/240 (70.0)	40/68 (58.7)	32/61 (53.4)	38/63 (63.0)	38/51 (76.4)	56/93 (62.9)	.05
Surfactant during first days of life	155/238 (64.0)	42/66 (62.9)	32/61 (51.2)	49/62 (78.1)	43/49 (86.8)	77/93 (80.9)	<.001
Attempted CPAP in the first 24 h of life	171/236 (73.2)	55/66 (84.8)	46/61 (76.5)	36/60 (62.8)	21/49 (43.7)	47/91 (54.0)	<.001
Ductus arterious treatment before day 10	41/237 (16.8)	13/67 (18.0)	7/58 (11.5)	29/62 (44.7)	18/51 (33.5)	41/91 (42.4)	<.001
Early neonatal infection	48/234 (20.0)	14/67 (20.4)	21/59 (35.5)	15/61 (24.1)	18/50 (36.5)	22/90 (23.9	.05
Late infection (after 72 h of life and before the stool collect)	85/229 (35.7)	10/65 (14.7)	14/60 (21.7)	35/60 (56.5)	41/52 (78.5)	75/90 (82.1)	<.001
Low volume of enteral nutrition at day 7	60/240 (24.4)	19/68 (26.9)	18/61 (29.4)	29/63 (44.3)	34/52 (65.0)	59/93 (62.4)	<.001
Gastrointestinal transit considered normal (at least 1 stool a day)	152/227 (67.5)	39/66 (59.9)	44/57 (77.9)	28/60 (46.8)	13/50 (25.9)	40/86 (46.8)	<.001
Practice of skin-to-skin contact during the first week of life	147/232 (64.2)	40/64 (63.2)	43/59 (73.9)	31/57 (55.2)	16/45 (34.8)	29/77 (39.2)	<.001
Breast milk during first week	139/239 (57.6)	43/68 (62.9)	46/61 (75.6)	40/60 (67.0)	33/52 (63.9)	59/93 (62.7)	.17
NICU's strategy^c^							
No intubation or extubation at day 1	95/240 (38.8)	28/68 (41.3)	27/61 (44.1)	23/63 (36.6)	15/52 (28.8)	28/93 (30.5)	.40
Sedation during the first week	161/240 (67.7)	46/68 (66.6)	44/61 (72.3)	46/63 (73.7)	43/52 (82.5)	65/93 (70.5)	.40
Medication to close ductus arteriosus before day 10	128/240 (54.3)	40/68 (58.3)	34/61 (56.3)	40/63 (63.8)	32/52 (62.2)	47/93 (50.5)	.58
Longer duration of primary antibiotherapy	89/240 (37.9)	18/68 (25.7)	27/61 (45.4)	21/63 (32.7)	16/52 (28.7)	26/93 (26.7)	.07
Longer duration of secondary antibiotherapy	113/240 (46.8)	23/68 (34.5)	21/61 (34.9)	21/63 (31.9)	15/52 (27.1)	43/93 (45.2)	.03
Low volume of enteral nutrition at day 7	60/240 (25.0)	29/68 (44.0)	11/61) (18.1	27/63 (43.1)	18/52 (35.0)	35/93 (36.3)	.001
Skin to skin during the first week	145/240 (60.3)	41/68 (60.6)	48/61 (79.4)	37/63 (59.5)	23/52 (42.9)	47/93 (49.8)	<.001
Direct breastfeeding during the first week	32/240 (12.4)	4/68 (5.7)	16/61 (25.6)	3/63 (4.3)	2/52 (3.4)	6/93 (6.8)	.002

Abbreviations: BMI, body mass index, calculated as weight in kilograms divided by height in meters squared; CPAP, continuous positive airway pressure; NICU, neonatal intensive care unit.

^a^ Percentages are weighted to account for differences in sampling process between gestational ages. Denominators vary according to the number of missing data for each variable.

^b^ Score based on Olsen curves.

^c^ Favorable strategy: the observed percentage was zero or greater than the expected percentage of newborn receiving the treatment or practice.

**Figure 2.**
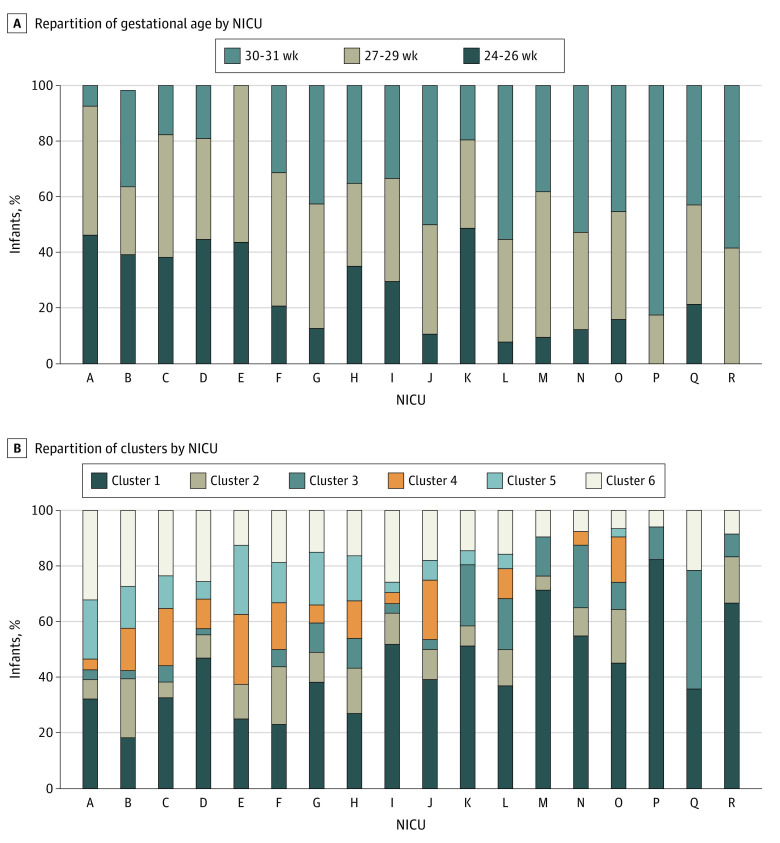
Repartition of Gestational Age and Microbiota Cluster in 18 Neonatal Intensives Care Units (NICUs) Enrolling More Than 10 Preterm Newborns Among 577 preterm newborns enrolled in the EPIFLORE study, 544 were hospitalized from 18 NICUs enrolling more than 10 preterm newborns each. Difference in gut microbiota composition among NICUs is not only dependent of gestational age (A). As an example, more than 60% of newborns hospitalized in NICU C belonged to clusters 4, 5, or 6 (B) and were considered immature, although more than 60% of them had a gestational age of more than 26 weeks. Conversely, less than 20% of the newborns of NICUs K stratified into clusters 4, 5, or 6, while 50% of them have a gestational age less than 27 weeks.

Among the 18 NICUs, we observed *E coli*_OTU7123, a significant variation of the repartition of clusters (Figure 2). We described an association between NICUs strategies and cluster belonging both before (Table 1) and after adjustment for cofounders, such as GA (eTable 3 in the Supplement). Favorable skin-to-skin strategy was associated with reduced risk of being in cluster 1, 5, or 6. No intubation or extubation at day 1 strategy was associated with a decrease risk of being in cluster 5 or 6. Favorable sedation strategy was associated with an increase risk of being in cluster 5 and 6. Lower volume of enteral nutrition strategy was associated with an increased risk of being in clusters 2, 4, 5, or 6. Thus, sedation during the first week and low volume of enteral nutrition was associated with increased risk of being in the more immature clusters after adjustment for confounders. No assisted ventilation at day 1, direct breastfeeding, and skin-to-skin practice was associated with decreased risk of being in the more immature clusters. For example, a 27-week preterm newborn born vaginally with a regular transit hospitalized in a NICU with favorable skin-to-skin practice had a median (IQR) risk of 26% (50%-74%) of being in clusters 4, 5, or 6, but being born by cesarean delivery without regular transit hospitalized in NICU with unfavorable skin-to-skin practice strategy had a median (IQR) risk of 63% (50%-74%) of being in clusters 4, 5, or 6. Moreover, in post hoc analysis, we observed an interaction between skin-to-skin as individual practice and as NICU strategy to increase the probability to be in the cluster 3, the more mature cluster (eFigure 5 in the Supplement).

**Figure 3.**
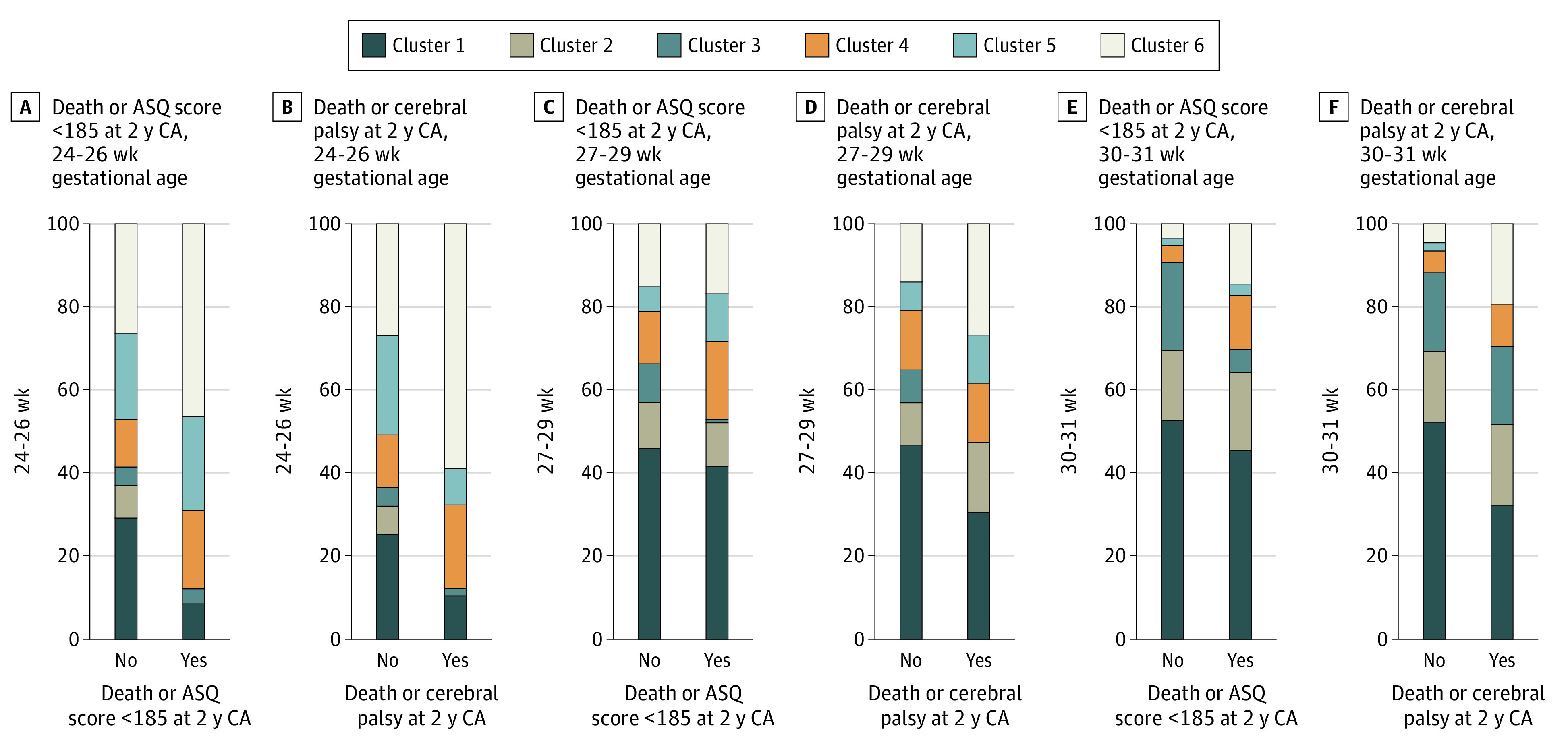
Repartition of Microbiota Clusters According to Gestational Age and 2-Year Outcome Two-year-outcome is defined by death or Ages and Stages questionnaire (ASQ) score less than 185 at 2 years of age (A, C, and E) and by death or cerebral palsy (B, D, and F) (result after multiple imputation). Percentages are weighted to take into account the differences in survey design between gestational age groups.

At 2-year corrected age for prematurity, 22 of the newborns had died and 555 were eligible for follow-up. Cerebral palsy information was obtained for 490 newborns (88.2% of eligible survivors) and ASQ data for 372 (67.0% of eligible survivors). Thus, the outcome of death or ASQ score more than 185 was available for 394 newborns and the outcome of death or cerebral palsy for 512 newborns (eFigure 1 in the Supplement). At follow-up, 65 of the newborns had an ASQ score less than 185 (17.1%; after multiple imputation, 20.6%): 14 newborns with cerebral palsy, 49 had an ASQ score less than 185 without cerebral palsy, and 2 had an ASQ score less than 185 without cerebral palsy data available. Death or ASQ score less than 185 was significantly associated with GA (mean [SD] GA, 27.6 [2.1] weeks and 28.4 [2.0] weeks, respectively; *P* = .01) and with cluster (49 of 84 [58.3%] and 118 of 375 [31.5%] newborns belonged to clusters 4, 5, or 6, respectively; *P* = <.001). We observed the same relation with death or cerebral palsy at 2 years (Figure 3; Table 2; eFigure 6 in the Supplement). Belonging to clusters 4, 5, or 6 was significantly associated with death or ASQ score of less than 185, before and after adjustment for GA, characteristics of neonates, and strategies (Table 2). Sensitivity analyses confirm this association (eTable 4 in the Supplement). Phylum Firmicutes, class Bacilli, and *S caprae*_OTU5825 were negatively correlated with GA (*r* = −0.27, *P* < .001; *r* = −0.37, *P* < .001; and *r* = −0.37, *P* < .001, respectively) and ASQ score (*r* = −0.12, *P* = .017; *r* = −0.14, *P* = .007; and *r* = −0.15, *P* = .14, respectively). Conversely, phylum Proteobacteria, class Gammaproteobacteria and *E coli*_OTU7123 were positively correlated with GA (*r* = 0.26, *P* < .001; *r* = 0.26, *P* < .001; and *r* = 0.16, *P* < .001, respectively) and ASQ score (*r* = 0.13, *P* = .009; *r* = 0.13, *P* = .009; and *r* = 0.12, *P* = .03, respectively).

**Table 2.** Association Between Microbiota Cluster and 2-Year Outcomes With Multiple Imputation^a^

Cluster	Average, No./No. (%)	*P* value	AOR (95% CI)	*P* value	AOR (95% CI)^b^	*P* value	AOR (95% CI)^c^	*P* value
**Death or ASQ score <185 at 2-y corrected age**								
Cluster 1	43/240 (18.5)	<.001	3.01 (0.83-10.89)	.001	2.79 (0.75-10.43)	.01	2.46 (0.67-9.07)	.03
Cluster 2	12/68 (19.2)		3.52 (0.87-14.26)		2.95 (0.68-12.82)		2.41 (0.57-10.25)	
Cluster 3	3/61 (6.0)		1 [Reference]		1 [Reference]		1 [Reference]	
Cluster 4	23/63 (37.4)		8.21 (2.02-33.35)		7.19 (1.67-30.87)		6.17 (1.46-26.00)	
Cluster 5	17/52 (34.6)		7.33 (1.77-30.35)		5.06 (1.14-22.51)		4.53 (1.02-20.06)	
Cluster 6	35/93 (37.0)		8.15 (2.14-31.08)		6.49 (1.61-26.13)		5.42 (1.36-21.58)	
**Death or cerebral palsy at 2-y corrected age**								
Cluster 1	14/240 (5.9)	<.001	1.33 (0.28-6.38)	.006	1.27 (0.24-6.75)	.01	1.31 (0.25-7.01)	.03
Cluster 2	6/68 (9.9)		2.53 (0.47-13.58)		2.27 (0.35-14.58)		2.06 (0.33-12.79)	
Cluster 3	2/61 (3.9)		1 [Reference]		1 [Reference]		1 [Reference]	
Cluster 4	9/63 (15.0)		3.37 (0.64-17.89)		3.57 (0.57-22.14)		2.97 (0.48-18.24)	
Cluster 5	5/52 (10.8)		1.99 (0.33-12.14)		2.59 (0.34-19.62)		2.45 (0.33-18.03)	
Cluster 6	23/93 (24.9)		6.10 (1.24-29.96)		7.93 (1.30-48.41)		5.94 (1.03-34.27)	

Abbreviations: AOR, adjusted for gestational age; ASQ, Ages and Stages questionnaire; NICU, neonatal intensive care unit.

^a^ Multiple imputation analysis. Odds ratio are estimated using mixed-effects logistic regression with a random hospital intercept. Percentages are weighted to account for differences in sampling process between gestational ages.

^b^ Adjusted for gestational age, maternal age, country of birth of the mother, mother level of education, birth weight Z-score, cesarean delivery, and individual therapeutics (surfactant, ductus arterious treatment before in the first 10 days of life, late neonatal infection, volume of enteral nutrition at day 7, gastrointestinal transit considered as regular, practice of skin-to-skin contact during the first week of life).

^c^ Adjusted for gestational age, maternal age, country of birth of the mother, mother level of education, birth weight Z-score, cesarean delivery, and all NICU's practice strategies.

## Discussion

In this multicenter prospective observational study, taxonomic composition of the gut microbiota was associated with characteristics of preterm newborns, including GA, birth weight Z-score, country of birth of the mother, birth by cesarean delivery, gastrointestinal transit at day 7, with individual treatment as low volume of enteral nutrition, and with practice strategies of NICUs such as no intubation or extubation at day 1, sedation during first week, low volume of enteral nutrition, and skin-to-skin practice during the first week after birth. Moreover, we highlight that the early composition of the gut microbiota was associated with 2-year outcomes, after adjustment for GA, characteristics of preterm newborns, and practices. To the best of our knowledge, these associations are observed for the first time.

Microbiota of very preterm newborns included in this study is characterized by a low diversity, which is consistent with previous studies.^6,8,30^ Six discrete microbiota-driven clusters can allow stratifying preterm newborns in the large present study that confirms an earlier smaller study.^31^ Interestingly, each cluster was driven by few dominant OTUs in the preterm-newborns nationwide cohort. Low bacterial load, *Staphylococcus*, and *Enterococcus* were associated with lower GA and reflected an immature microbiota. By contrast *E coli*, which reflects a more mature microbiota as suggested in 2 dynamic studies, in term newborns^32^ and preterm ones,^33^ is associated with higher GA. Colonization by bifidobacteria was scarce in these very preterm newborns, as previously described.^34^

Two-year nonoptimal outcome is associated with the more immature microbiota. This is in line with the described association between low gestational age and neurodevelopmental delay in the EPIPAGE 2 cohort.^23^ While composition of gut microbiota could solely be a biological marker that reflects immaturity and severity of illness, this association persists after adjustment for characteristics of preterm newborns and received treatments, which suggests a more contributory role of the gut microbiota in the outcome. Indeed, the past 2 decades have been characterized by discoveries about the link between gut microbiota and brain function. Some evidence seems to indicate that a healthy microbiota early in life could play a key role for a correct neurodevelopment though metabolites or other microbiota-derived molecules.^35^ This question must be studied in animal models to investigate the potential impact of specific bacteria or microbiota profiles on long-term neurodevelopment in newborns.^36^

Practice strategies of NICUs, such as less sedation during the first week, no intubation or early extubation at day 1, and skin-to-skin practice during the first week, influenced the gut microbiota composition toward a more mature profile, independently of the GA. These associations between practice strategies and microbiota are observed outside of nutritional interventions. This has been possible because EPIFLORE is an ancillary study of a large nationwide cohort.^19^ Interventional randomized clinical trials will be required to confirm the hypothesis that modification of practices can modulate gut microbiota and influence the 2-year outcome.

### Limitations and Strengths

This study has limitations. The main limitations of this study are uncontrolled confounding bias or reverse causation. Another limitation is the absence of environmental sampling inside of NICUs.^15,37^ Practices and environment are probably intertwined^38^ and must be studied in future prospective multicenter studies. In this study, we cannot eliminate the hypothesis that observed differences are associated or because of a different bacteriological environment, but always associated with different practices. Microbiota analysis of only 1 sample around week 4 after birth, which does not take into account dynamic changes of microbiota during the first year of life, constitutes another limitation. Post hoc analysis must be confirmed in future multicenter studies.

However, the present study has major strengths. First, the multicenter study is integrated in the EPIPAGE 2 study, a population-based cohort that enrolled newborns born prematurely in France in 2011. This cohort made it possible to accurately characterize the strategies of the NICUs, with a very accurate description of therapeutics during hospitalization. Second, the number of analyzed newborns consequently belongs to numerous NICUs all over the country and validates these results while allowing their extension. Hence, despite the limitations related to the observational nature of this study, these unique results are in favor of an association between practices strategies, the early gut microbiota establishment in very preterm newborns, and newborns’ outcomes.

## Conclusions

In this study, composition of the gut microbiota of preterm newborn at 4 weeks after birth was associated with 2-year outcomes and varied according to intertwined associations of GA, perinatal characteristics, individual treatments received, and NICU therapeutic strategies. These findings suggest microbiota as a new noninvasive biomarker. Moreover, future intervention trials should evaluate whether either modifying strategies, such as promoting enteric nutrition, reducing sedation use, reducing the use of assisted ventilation, or promoting skin-to-skin practice, microbiota-based therapeutics, or both, could consequently improve prognosis of very preterm newborns.

## Notes

Supplement. **eMethods.** Neonatal intensive care units’ strategies **eTable 1.** Comparison between preterm infants with and without faecal collect among eligible preterm infants **eTable 2.** Characteristics of preterm infants, their mothers and the individual treatments received by preterm infants according to the cluster describing microbiota at one month after birth **eTable 3.** Characteristics of preterm infants, their mothers and the treatment strategies of the NICU where the preterm infant was hospitalized at day 7 according to the cluster describing microbiota at one month after birth **eTable 4.** Association between microbiota cluster and 2 years-outcome: Complete cases analysis **eFigure 1.** Flow chart **eFigure 2.** Difference between observed and expected percent of eight practices in 60 neonatal intensive care units participating in EPIPAGE study **eFigure 3.** Practice strategies of the 18 neonatal intensive care units (NICU) participating to EPIFLORE project where more 10 infants were hospitalized **eFigure 4.** Principal coordinate analysis (PCoA) plots of the Bray-Curtis distance at the genus (A) and OTUs (B) taxonomic profiles **eFigure 5.** Skin-to-skin practice as risk factor for cluster 3, the most mature microbiota **eFigure 6.** Repartition of microbiota clusters according to gestational age and 2 year-outcome (complete cases)
