# Supplementary material for: Assessment of Neonatal Intensive Care Unit Practices and Preterm Newborn Gut Microbiota and 2-Year Neurodevelopmental Outcomes
Source: JAMA Netw Open. 2020 Sep 23;3(9):e2018119. doi: 10.1001/jamanetworkopen.2020.18119 (PMC7512059; doi:10.1001/jamanetworkopen.2020.18119)
Supplement: Supplement. — eMethods. Neonatal intensive care units’ strategies eTable 1. Comparison between preterm infants with and without faecal collect among eligible preterm infants eTable 2. Characteristics of preterm infants, their mothers and the individual treatments received by preterm infants according to the cluster describing microbiota at one month after birth eTable 3. Characteristics of preterm infants, their mothers and the treatment strategies of the NICU where the preterm infant was hospitalized at day 7 according to the cluster describing microbiota at one month after birth eTable 4. Association between microbiota cluster and 2 years-outcome: Complete cases analysis eFigure 1. Flow chart eFigure 2. Difference between observed and expected percent of eight practices in 60 neonatal intensive care units participating in EPIPAGE study eFigure 3. Practice strategies of the 18 neonatal intensive care units (NICU) participating to EPIFLORE project where more 10 infants were hospitalized eFigure 4. Principal coordinate analysis (PCoA) plots of the Bray-Curtis distance at the genus (A) and OTUs (B) taxonomic profiles eFigure 5. Skin-to-skin practice as risk factor for cluster 3, the most mature microbiota eFigure 6. Repartition of microbiota clusters according to gestational age and 2 year-outcome (complete cases) [file jamanetwopen-e2018119-s001.pdf]

Supplementary Online Content

Rozé JC, Ancel PY, Marchand-Martin L, et al; EPIFLORE Study Group. Assessment of neonatal intensive care unit practices and preterm newborn gut microbiota and 2-year neurodevelopmental outcomes. *JAMA Netw Open*. 2020;3(9):e2018119. doi:10.1001/jamanetworkopen.2020.18119

eMethods. Neonatal intensive care units' strategies

eTable 1. Comparison between preterm infants with and without faecal collect among eligible preterm infants

eTable 2. Characteristics of preterm infants, their mothers and the individual treatments received by preterm infants according to the cluster describing microbiota at one month after birth

eTable 3. Characteristics of preterm infants, their mothers and the treatment strategies of the NICU where the preterm infant was hospitalized at day 7 according to the cluster describing microbiota at one month after birth

eTable 4. Association between microbiota cluster and 2 years-outcome: Complete cases analysis

eFigure 1. Flow chart

eFigure 2. Difference between observed and expected percent of eight practices in 60 neonatal intensive care units participating in EPIPAGE study

eFigure 3. Practice strategies of the 18 neonatal intensive care units (NICU) participating to EPIFLORE project where more 10 infants were hospitalized

eFigure 4: Principal coordinate analysis (PCoA) plots of the Bray-Curtis distance at the genus (A) and OTUs (B) taxonomic profiles

eFigure 5. Skin-to-skin practice as risk factor for cluster 3, the most mature microbiota

eFigure 6. Repartition of microbiota clusters according to gestational age and 2 year-outcome (complete cases)

© 2020 Rozé JC et al. JAMA Network Open.

eMethod

- **Nutrition strategies of NICUs**

We characterized 8 strategies for each neonatal intensive care units (NICUs) as previously described (1), no intubation or extubation during the first 24 hours after birth, use of sedation during the first week after birth, direct breastfeeding during the first week after birth, skin to skin during the first week after birth, and treatment of ductus arteriosus during the first ten days after birth, speed of progression of enteral feeding during the first week after birth, duration of primary antibiotherapy started during the first 48 hours after birth and duration of secondary antibiotherapy.

- **Strategies concerning received or not received treatments**

For the first 5 strategies concerning received or not received treatments, a probability to receive each treatment for each preterm infant was calculated by logistic regression, according to gestational age, birth weight Z-score, sex, inborn or outborn, mode of birth, type of prematurity, single or multiple pregnancy, antenatal corticosteroid administration, Apgar score, number of administered doses of surfactant, birth nationality of mother and socioeconomic level of the parents using EPIPAGE2 database. From the average of the probabilities of receiving the treatment for children from the same NICU, we calculated an expected percentage to have this strategy applied in this NICU. If the observed difference in percentage was zero or greater than the expected percentage, the NICU strategy was considered favourable to the application of this strategy. If the difference was negative, the strategy was considered unfavourable.

No intubation or extubation during the first 24 hours after birth

For each infant$_i$ a probability, p(Infant$_i$), to be not intubated or extubated during the first 24 hours after birth was calculated by logistic regression according the characteristics of the preterm infant and his parents. Then we calculated an expected percentage of to be not intubated or extubated during the first 24 hours after birth for each NICU, expressed as %, according to the following formula:

Expected percentage of infants not intubated or extubated during the first 24 hours after birth of one NICU

$$= 100 \times (\Sigma_0^N (p(Infant_i)) / \text{Number of infants of this NICU}$$

Then we calculated the difference between the observed and the expected percentage of infants not intubated or extubated during the first 24 hours after birth for each unit. If the difference $\geq$ or < zero, the NICU was considered favourable or not favourable to limit intubation, respectively.

Use of sedation during the first week after birth

For each infant$_i$ a probability, *p(Infant$_i$),* to receive sedation during the first week after birth was calculated by logistic regression according the characteristics of the preterm infant and his parents. Then we calculated an expected percentage of to be sedated during the first week after birth for each NICU, expressed as %, according to the following formula :

Expected percentage of infants sedated during the first week after birth of one NICU

$$= 100 \times (\Sigma_0^N (p(Infant_i)) / \text{Number of infants of this NICU}$$

Then we calculated the difference between the observed and the expected percentage of infants receiving a sedation during the first week after birth for each unit. If the difference $\geq$ or < zero, the NICU was considered favourable or not favourable to use of sedation, respectively.

Direct-Breastfeeding policy

For each infant$_i$ a probability, *p(Infant$_i$),* to be partially direct-breastfed during the first week after birth was calculated by logistic regression according to gestational age, birth weight Z-score, sex, birth Nationality of mother, level of education. Then we calculated an expected percentage of partial direct-breastfeeding during the first week for each NICU, expressed as %, according to the following formula :

Expected percentage of infants partially direct-breastfed during the first week after birth of one NICU

$$=100 \times (\Sigma_0^N (p(Infant_i)) / \text{Number of infants of this NICU}$$

Then we calculated the difference between the observed and the expected percentage of infants direct breastfed during the first week after birth for each unit. If the difference $\geq$ or < zero, the NICU was considered as favourable to direct breastfeeding or not favourable, respectively.

Skin to skin during the first week after birth

For each infant$_i$ a probability, *p(Infant$_i$),* to be in skin to skin contact with at least one of the parents during the first week of life after birth was calculated by logistic regression according the characteristics of the preterm infant. Then we calculated an expected percentage of to be in skin-to-skin contact during the first week after birth for each NICU, expressed as %, according to the following formula :

Expected percentage of infants in skin to skin contact during the first week after birth of one NICU

© 2020 Rozé J-C et al. JAMA Network Open.

$= 100 \times (\Sigma_0^N (p(Infant_i)) /$ Number of infants of this NICU

Then we calculated the difference between the observed and the expected percentage of infants being in skin to skin contact during the first week after birth for each unit. If the difference $\geq$ or < zero, the NICU was considered as favourable to skin to skin contact or not favourable, respectively.

Treatment of ductus arteriosus during the first ten days after birth

For each infant$_i$ a probability, *p(Infant$_i$),* to be treated by Ibuprofen during the first 10 days after birth was calculated by logistic regression according the characteristics of the preterm infant. Then we calculated an expected percentage of to be to be treated by Ibuprofen during the first 10 days after birth for each NICU, expressed as %, according to the following formula :

Expected percentage of infants to be treated by Ibuprofen during the first 10 days after birth of one NICU

$= 100 \times (\Sigma_0^N (p(Infant_i) /$ Number of infants of this NICU

Then we calculated the difference between the observed and the expected percentage of infants receiving Ibuprofen during the first 10 days after birth for each unit. If the difference $\geq$ or < zero, the NICU was considered as favourable to close ductus arteriosus during the first 10 days after birth or not favourable, respectively.

- **Strategies concerning duration or quantity of treatment**

Longer duration of primary antibiotic treatments

Concerning primary antibiotic treatment started during the first 48 hours of life, an expected duration was calculated with an individual 95% confidence interval (CI), using linear regression according to explicative variables: type of prematurity, confirmed early neonatal infection and neonatal characteristics. If the observed duration was more than upper limit of the 95% CI, the duration of antibiotic treatment was considered as longer than the expected duration. As for other strategies, an expected probability for each infant was calculated by logistic regression. Expected percentage of infants with a longer duration of primary antibiotherapy of one NICU

$= 100 \times (\Sigma_0^N (p(Infant_i)) /$ Number of infants of this NICU

Then we calculated the difference between the observed and the expected percentage of infants with longer duration of primary antibiotic treatment for each unit. If the difference $\geq$ or < zero, the NICU was considered as favourable to longer duration of primary antibiotic therapy or not favourable, respectively.

Longer duration of secondary antibiotic treatments

Concerning secondary antibiotic treatment administered, an expected duration was calculated with an individual 95% confidence interval (CI), using linear regression according to explicative variables: type of prematurity, confirmed secondary infection and neonatal characteristics. If the observed duration was more than upper limit of the 95% CI, the duration of antibiotic treatment was considered as longer than the expected duration. As for other strategies, an expected probability for each infant was calculated by logistic regression. Expected percentage of infants with a longer duration of secondary antibiotic therapy of one NICU

$= 100 \times (\Sigma_0^N (p(Infant_i)) /$ Number of infants of this NICU

Then we calculated the difference between the observed and the expected percentage of infants with longer duration of secondary antibiotic treatment for each unit. If the difference $\geq$ or < zero, the NICU was considered as favourable to longer duration of secondary antibiotic therapy or not favourable, respectively.

Speed of progression of enteral feeding

We first calculated an expected enteral volume at day 7 with a 95% confidence interval (95%CI) for each infant, according to gestational age, birth weight Z-score and regularity of intestinal transit during the first 7 days. A low volume of enteral feeding was defined as a volume less than the lower limit of the 95%CI. As for other strategies, an expected probability to receive a low enteral volume at day 7 was calculated for each infant by logistic regression. Expected percentage of infants with a low enteral volume at day 7 of one NICU

$= 100 \times (\Sigma_0^N (p(Infant_i)) /$ Number of infants of this NICU

Then we calculated the difference between the observed and the expected percentage of infants with low enteral volume at day 7 for each unit. If the difference $\geq$ or < zero, the NICU was considered as favourable to provide low enteral feeding during the first 7 days after birth or not favourable, respectively.

© 2020 Rozé J-C et al. JAMA Network Open.

eTable 1. Comparison between preterm infants with and without faecal collect among eligible preterm infants.

	Preterm with fecal collect at 1 month old		Eligible preterm without fecal collect*		
	n=577		n=529		p-value
**Gestational age**					
24-26 weeks	145/577	20.0	101/529	14.9	<.001
27-29 weeks	226/577	41.9	171/529	34.0	
30-31 weeks	206/577	38.2	257/529	51.1	
**Maternal characteristics**					
Maternal age					
<25 years	96/577	16.6	113/529	21.5	.12
25-34 years	342/577	59.4	296/529	55.4	
≥35 years	139/577	23.9	120/529	23.1	
Mother born outside France					
No	420/577	72.9	345/529	65.6	.02
Yes	154/577	26.6	176/529	33.0	
Missing	3/577	0.5	8/529	1.4	
Mother level of education					
< Higher secondary school	151/577	26.6	140/529	27.1	.17
Higher secondary school	108/577	18.5	104/529	19.5	
High school diploma +1 +2	120/577	20.9	83/529	15.4	
> High school diploma +3	145/577	25.3	145/529	27.4	
Missing	53/577	8.6	57/529	10.6	
Multiple pregnancy	197/577	34	160/529	30.2	.17
**Neonatal factors**					
Male	300/577	52.0	274/529	51.8	.95
Birth weight Z-score†, mean(sd)	577	0.2 (1.0)	529	0.1 (1.1)	.12
Apgar score <7 at 5 minutes					
No	449/577	78.7	400/529	76.3	.07
Yes	94/577	15.7	108/529	19.9	
Missing	34/577	5.6	21/529	3.8	
Gastrointestinal transit considered normal (at least one stool a day)					
No	230/577	38.8	155/529	28.3	<.001
Yes	316/577	55.7	320/529	61.6	
Missing	31/577	5.5	54/529	10.2	
Severe neonatal morbidities‡					
No	452/577	79.8	429/529	82.1	.57
Yes	102/577	16.5	79/529	14.2	
Missing	23/577	3.7	21/529	3.7	
Died in neonatal intensive care unit	18/577	2.7	18/529	3.1	.64
**2 years corrected age**					
Cerebral palsy					
No	459/559	82.1	72/511	14.2	.58
Yes	31/559	5.6	414/511	81.0	
Missing	69/559	12.3	25/511	4.8	
Ages and Stages Questionnaire (ASQ) score < 185					
No	307/388	79.3	251/330	77.0	.52
Yes	65/388	16.4	67/330	19.5	
Missing	16/388	4.3	12/330	3.6	

Complete cases analysis. Data are number of events/number in group (percentages). Percentages are weighted to take into account the differences in survey design between gestational age groups. Denominators vary according to the number of missing data for each variable.

* Eligible survivors at J21 non included in EPIFLORE project n=386 or included without fecal collect 1 month old n=143).

† Score based on Olsen curves.

‡ Severe neonatal morbidity was defined as severe bronchopulmonary dysplasia or necrotizing enterocolitis stage 2-3 or severe retinopathy of prematurity stage >3 or any of the following severe cerebral abnormalities on cranial ultrasonography: intraventricular haemorrhage grade III or IV or cystic periventricular leukomalacia (Ancel 2015).

© 2020 Rozé J-C et al. JAMA Network Open.

eTable 2. Characteristics of preterm infants, their mothers and the individual treatments received by preterm infants according to the cluster describing microbiota at one month after birth.

	Cluster 1	Cluster 2	Cluster 4	Cluster 5	Cluster 6	
	**vs Cluster 3** (n=61)					
	n=240	n=68	n=63	n=52	n=93	p-value*
**Gestational age**						
24-26 weeks	2.42 (0.74-7.97)	2.08 (0.50-8.72)	12.60 (2.89-54.86)	66.29 (11.79-372.84)	20.23 (4.97-82.34)	<.001
27-29 weeks	2.34 (1.08-5.06)	1.96 (0.77-5.01)	6.74 (2.32-19.57)	8.77 (2.11-36.50)	6.17 (2.14-17.79)	
30-31 weeks	1	1	1	1	1	
**Maternal characteristics**						
Country of birth of the mother						
France	1	1	1	1	1	.03
North Africa countries	2.20 (0.60-8.08)	6.81 (1.82-25.52)	1.14 (0.20-6.43)	3.05 (0.61-15.35)	2.49 (0.57-10.92)	
Other African countries	2.41 (0.72-8.00)	0.33 (0.03-3.16)	1.63 (0.37-7.12)	2.78 (0.62-12.51)	1.85 (0.46-7.43)	
Other	1.84 (0.48-7.00)	1.13 (0.21-6.14)	2.65 (0.55-12.63)	4.98 (0.86-28.91)	1.71 (0.32-9.25)	
**Neonatal factors**						
Birth weight Z-score†, mean(sd)	1.06 (0.76-1.46)	0.69 (0.46-1.03)	0.64 (0.42-0.99)	0.82 (0.51-1.33)	0.79 (0.53-1.18)	.02
Cesarean section	2.73 (1.42-5.26)	1.36 (0.62-3.00)	1.98 (0.85-4.60)	7.23 (2.58-20.21)	2.67 (1.17-6.09)	.002
**Neonatal factors and individual therapeutics**						
Surfactant during first days of life	1.00 (0.50-2.00)	1.12 (0.47-2.66)	1.08 (0.41-2.83)	1.06 (0.31-3.58)	0.77 (0.30-1.95)	.97
Ductus arterious treatment before in the first ten days of life	1.86 (0.67-5.17)	2.25 (0.69-7.33)	3.96 (1.28-12.26)	1.22 (0.37-4.10)	2.47 (0.84-7.27)	.08
Late infection (after 72 hours of life and before the stool collect)	1.42 (0.57-3.56)	0.59 (0.18-1.94)	1.33 (0.47-3.78)	2.30 (0.78-6.80)	3.98 (1.50-10.51)	<.001
Low volume of enteral nutrition at day 7	0.44 (0.19-0.99)	0.37 (0.13-1.00)	0.41 (0.15-1.09)	0.91 (0.31-2.66)	0.96 (0.39-2.41)	.03
Gastrointestinal transit considered normal (at least one stool a day)	0.56 (0.26-1.19)	0.41 (0.17-0.97)	0.35 (0.14-0.87)	0.20 (0.07-0.56)	0.53 (0.22-1.23)	.03
Practice of skin-to-skin contact during the first week of life	0.81 (0.39-1.65)	0.76 (0.33-1.76)	0.78 (0.33-1.87)	0.45 (0.16-1.24)	0.49 (0.18-1.36)	.36
Mother's milk during first week	0.37 (0.19-0.73)	0.50 (0.22-1.12)	0.57 (0.24-1.33)	0.43 (0.17-1.11)	0.40 (0.18-0.91)	.10

Multiple imputation analysis. Mixed-effects logistic regression with a random hospital intercept. The models included all table covariates.

* Global chi squared test.

† Score based on Olsen curves.

© 2020 Rozé J-C et al. JAMA Network Open.

eTable 3. Characteristics of preterm infants, their mothers and the treatment strategies of the NICU where the preterm infant was hospitalized at day 7 according to the cluster describing microbiota at one month after birth.

	Cluster 1	Cluster 2	Cluster 4	Cluster 5	Cluster 6	
	**vs Cluster 3** (n=61)					
	n=240	n=68	n=63	n=52	n=93	p-value*
**Gestational age**						
24-26 weeks	3.30 (1.12-9.69)	3.14 (0.84-11.69)	39.22 (10.37-148.40)	304.99 (62.04-1499.4)	94.06 (25.69-344.3)	<.001
27-29 weeks	2.29 (1.15-4.57)	2.09 (0.90-4.86)	9.81 (3.71-25.98)	14.84 (3.95-55.75)	10.69 (4.05-28.25)	
30-31 weeks	1	1	1	1	1	
**Maternal characteristics**						
Country of birth of the mother						
France	1	1	1	1	1	026
North Africa countries	1.77 (0.48-6.51)	6.11 (1.60-23.40)	0.81 (0.14-4.60)	2.08 (0.40-10.72)	1.69 (0.38-7.56)	
Other African countries	1.89 (0.56-6.38)	0.29 (0.03-2.87)	1.13 (0.25-5.16)	2.21 (0.48-10.27)	1.33 (0.31-5.63)	
Other	1.85 (0.49-7.01)	1.43 (0.26-7.88)	2.80 (0.56-13.89)	4.31 (0.72-25.67)	1.53 (0.27-8.68)	
**Neonatal factors**						
Birth weight Z-score†, mean(sd)	1.17 (0.85-1.60)	0.82 (0.56-1.21)	0.69 (0.46-1.04)	0.70 (0.44-1.13)	0.66 (0.44-0.98)	.001
Cesarean section	3.01 (1.55-5.86)	1.51 (0.67-3.43)	2.40 (1.00-5.75)	8.97 (3.17-25.34)	3.30 (1.43-7.62)	<.001
**NICU's strategie‡**						
No intubation or extubation at day 1	0.71 (0.28-1.77)	0.56 (0.19-1.66)	0.41 (0.13-1.28)	0.21 (0.06-0.78)	0.19 (0.06-0.62)	.03
Sedation during the first week	1.89 (0.63-5.62)	1.49 (0.43-5.21)	2.31 (0.64-8.37)	10.55 (2.28-48.87)	4.62 (1.32-16.18)	.02
Medication to close ductus arteriosus before day 10	0.96 (0.46-1.97)	0.71 (0.30-1.66)	0.94 (0.38-2.32)	0.89 (0.33-2.45)	0.62 (0.26-1.48)	.74
Longer duration of primary antibiotherapy	0.93 (0.42-2.06)	0.84 (0.31-2.24)	1.03 (0.36-2.95)	0.56 (0.16-1.94)	0.45 (0.15-1.32)	.57
Longer duration of secondary antibiotherapy	2.06 (0.76-5.62)	0.77 (0.24-2.48)	0.71 (0.22-2.31)	0.75 (0.20-2.78)	1.64 (0.52-5.20)	.02
Low volume of enteral nutrition at day 7	1.97 (0.63-6.18)	4.81 (1.38-16.77)	5.96 (1.64-21.64)	10.48 (2.48-44.29)	7.28 (2.03-26.18)	.001
Skin to skin during the first week	0.38 (0.16-0.90)	0.47 (0.17-1.30)	0.36 (0.12-1.06)	0.14 (0.04-0.48)	0.33 (0.11-0.95)	.07
Direct breastfeeding during the first week	0.76 (0.24-2.35)	0.40 (0.08-1.98)	0.25 (0.04-1.45)	0.23 (0.03-1.96)	0.43 (0.09-2.14)	.52

Abbreviations: NICU, neonatal intensive care unit.

Multiple imputation analysis. Mixed-effects logistic regression with a random hospital intercept. The models included all table covariates.

* Global chi squared test.

† Score based on Olsen curves.

‡ Favorable strategie, the observed percentage was zero or greater than the expected percentage of infant receiving the treatment or practice.

© 2020 Rozé J-C et al. JAMA Network Open.

eTable 4. Association between microbiota cluster and 2 years-outcome: Complete cases analysis.

	n/N	%	p-value	weighted %	p-value	AdjOR1	p-value	AdjOR2	p-value
**Death or ASQ score < 185 at 2 years corrected age**									
Cluster 1	24/162	14.8	<.001	14.9	<.001	3.04 (0.68 to 13.70)	.065	3.06 (0.68 to 13.66)	.01
Cluster 2	10/50	20.0		20.5		4.95 (1.00 to 24.51)		4.59 (0.94 to 22.48)	
Cluster 3	2/40	5.0		4.4		1		1	
Cluster 4	11/37	29.7		28.9		6.40 (1.24 to 33.15)		6.50 (1.28 to 32.91)	
Cluster 5	14/39	35.9		36.1		8.64 (1.67 to 44.65)		8.12 (1.61 to 40.93)	
Cluster 6	26/66	39.4		37.5		10.49 (2.18 to 50.53)		9.46 (1.99 to 44.87)	
**Death or cerebral palsy at 2 years corrected age**									
Cluster 1	11/210	5.2	<.001	5.2	<.001	2.70 (0.33 to 22.12)	.003	2.82 (0.35 to 22.77)	.005
Cluster 2	6/62	9.7		10.0		6.25 (0.69 to 56.37)		5.69 (0.65 to 49.94)	
Cluster 3	1/57	1.8		1.8		1		1	
Cluster 4	8/52	15.4		14.6		7.09 (0.79 to 63.54)		7.43 (0.85 to 65.06)	
Cluster 5	5/46	10.9		11.5		5.38 (0.54 to 53.46)		4.80 (0.50 to 45.84)	
Cluster 6	22/85	25.9		25.0		15.00 (1.79 to 125.77)		13.80 (1.68 to 113.13)	

Abbreviation: NICU, neonatal intensive care unit; ASQ, ages and stages questionnaire.

Odds ratio are estimated using mixed-effects logistic regression with a random hospital intercept. Percentages are weighted to take into account the differences in survey design between gestational age groups.

AdjOR1 : adjusted for gestational age by week.

AdjOR2 : adjusted for gestational age, continuous variable.

© 2020 Rozé J-C et al. JAMA Network Open.

eFigure 1. Flow chart

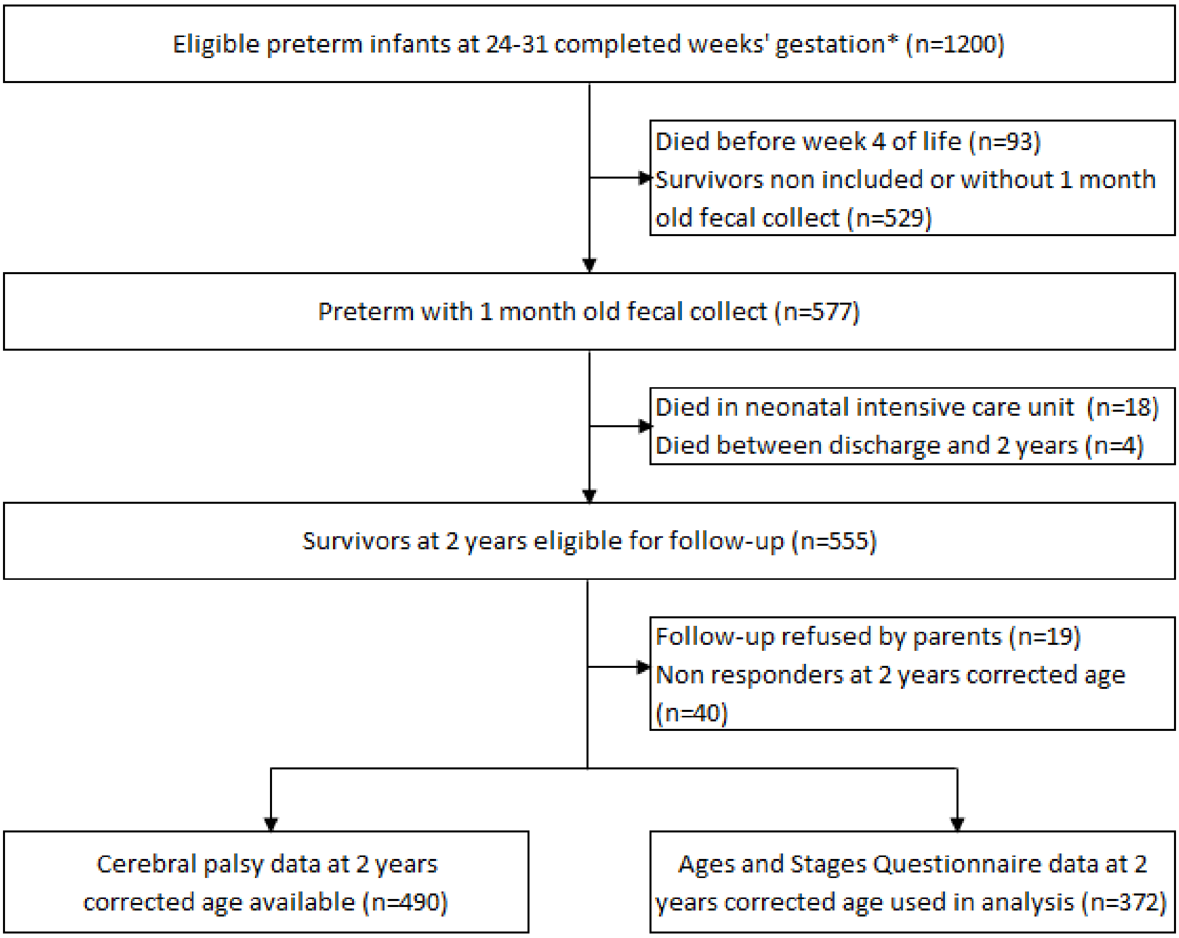

*Included in EPIPAGE2 and hospitalized in NICUs participating in EPIFLORE study.
** ASQ completed between 22 and 26 months'corrected age
NICUs: Neonatal intensive care units; ASQ: Ages and Stages Questionnaire.

© 2020 Rozé J-C et al. JAMA Network Open.

eFigure 2. Difference between observed and expected percent of eight practices in 60 neonatal intensive care units participating in EPIPAGE study

All neonatal intensive care units (NICUs) participating in EPIPAGE 2 study are represented. The white open circles represent NICUs that did not participate in EPIFLORE study. The black solid circles represent the NICUs participating to EPIFLORE study. On the Y axis, differences between observed and expected percentage of infants receiving the treatment in each NICU are represented, NICU by NICU.

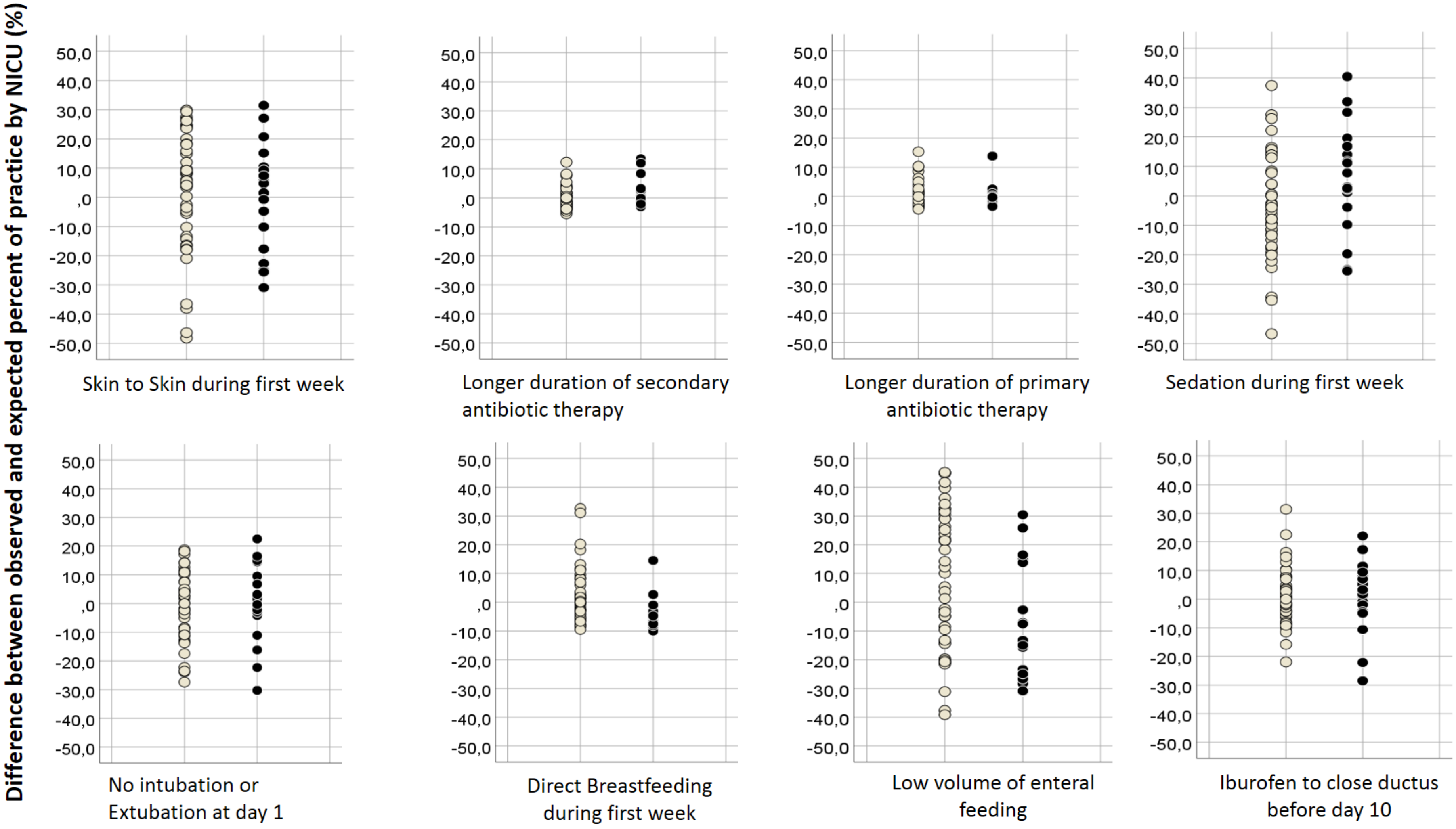

© 2020 Rozé J-C et al. JAMA Network Open.

eFigure 3: Practice strategies of the eighteen neonatal intensive care units (NICU) participating to EPIFLORE project where more 10 infants were hospitalized.

Each NICU participating to EPIFLORE study is represented by a letter. For each strategy, the colour of the corresponding square is green if the strategy of the concerned NICU is favourable, red if it is not favourable to the concerned strategy.

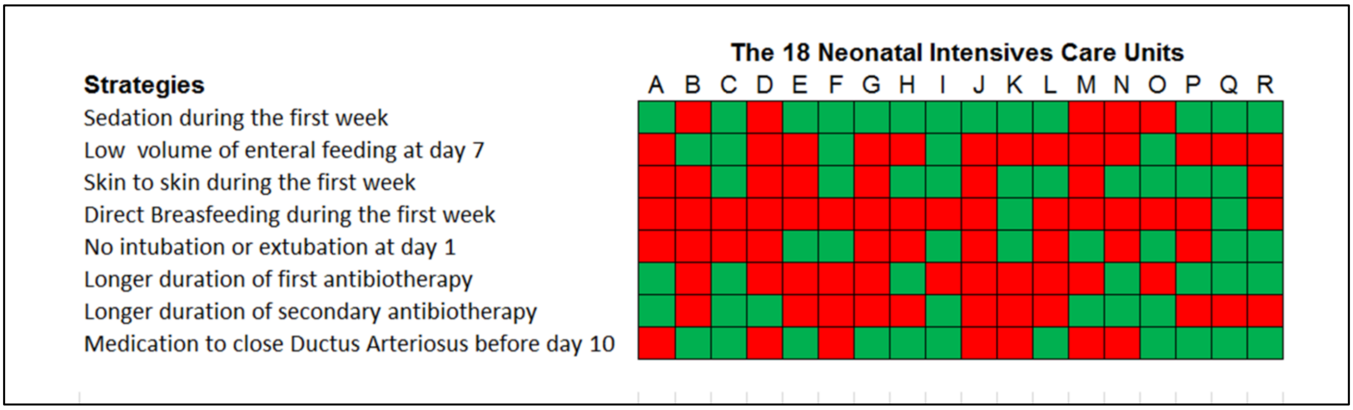

eFigure 4: Principal coordinate analysis (PCoA) plots of the Bray-Curtis distance at the genus (A) and OTUs (B) taxonomic profiles.

The five clusters are represented by different colours. For both plots, Comp1= 28% and Comp2= 14%

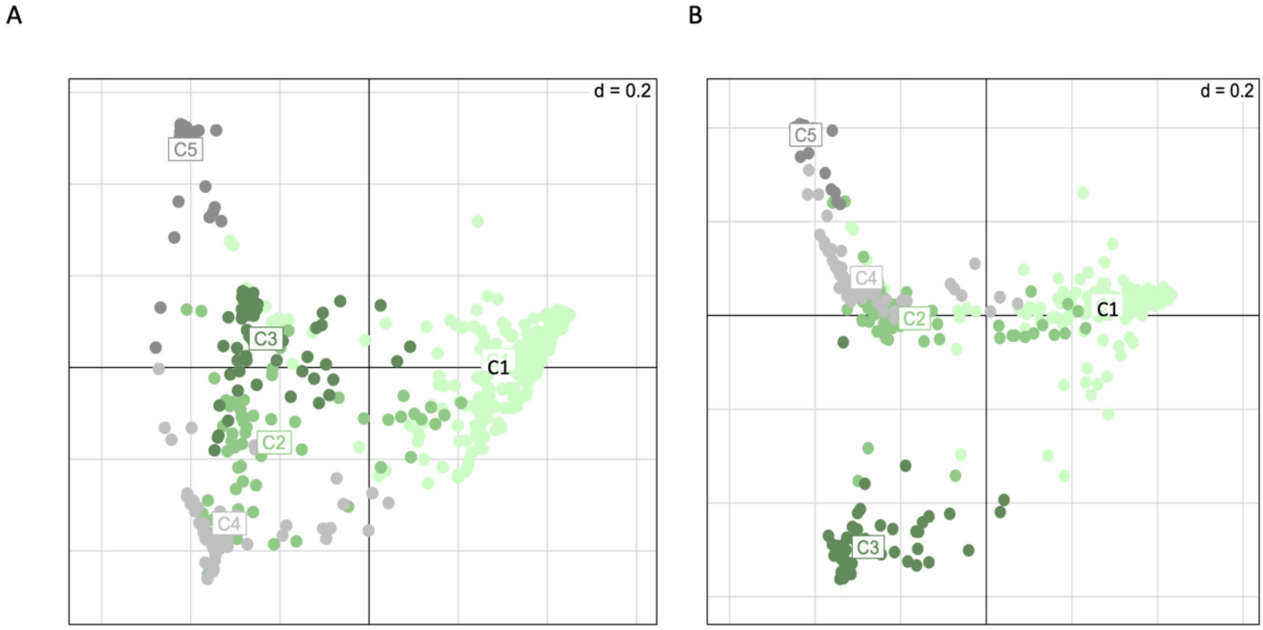

© 2020 Rozé J-C et al. JAMA Network Open.

eFigure 5. Skin-to-skin practice as risk factor for cluster 3, the most mature microbiota.

Percentage of preterm infants with a more mature microbiota (cluster 3) during the first month after birth depending on whether the preterm infants has been in skin to skin contact with parents during the first week after birth among NICUs with a strategy favourable or not favourable to skin to skin contact with parents. Adjusted odds ratios represent the probability to be in cluster 3 adjusted on propensity score to be in skin to skin contact during the first week after birth. We observed interaction between skin to skin contact with parents and NICU's strategy concerning skin to skin (p=0.01).

198

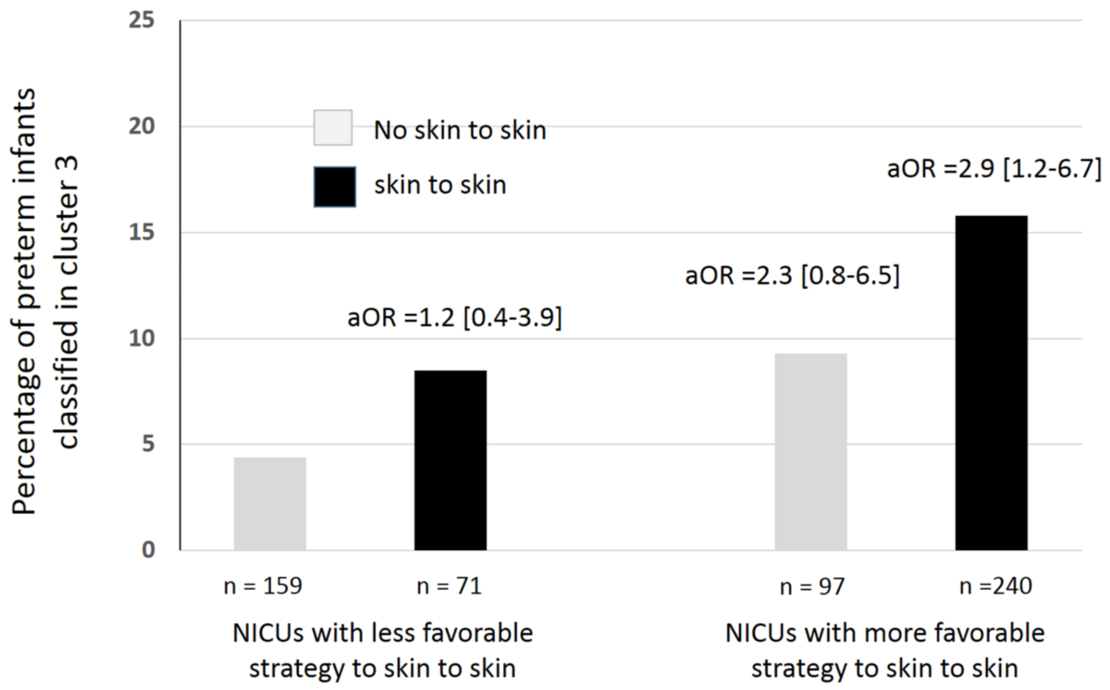

© 2020 Rozé J-C et al. JAMA Network Open.

eFigure 6. Repartition of microbiota clusters according to gestational age and 2 year-outcome (complete cases).

Two year-outcome is defined by death or Age and Stages Questionaries' score less than 185 at 2 years on the part A of the figure, and by death or cerebral palsy on the part B of the figure.

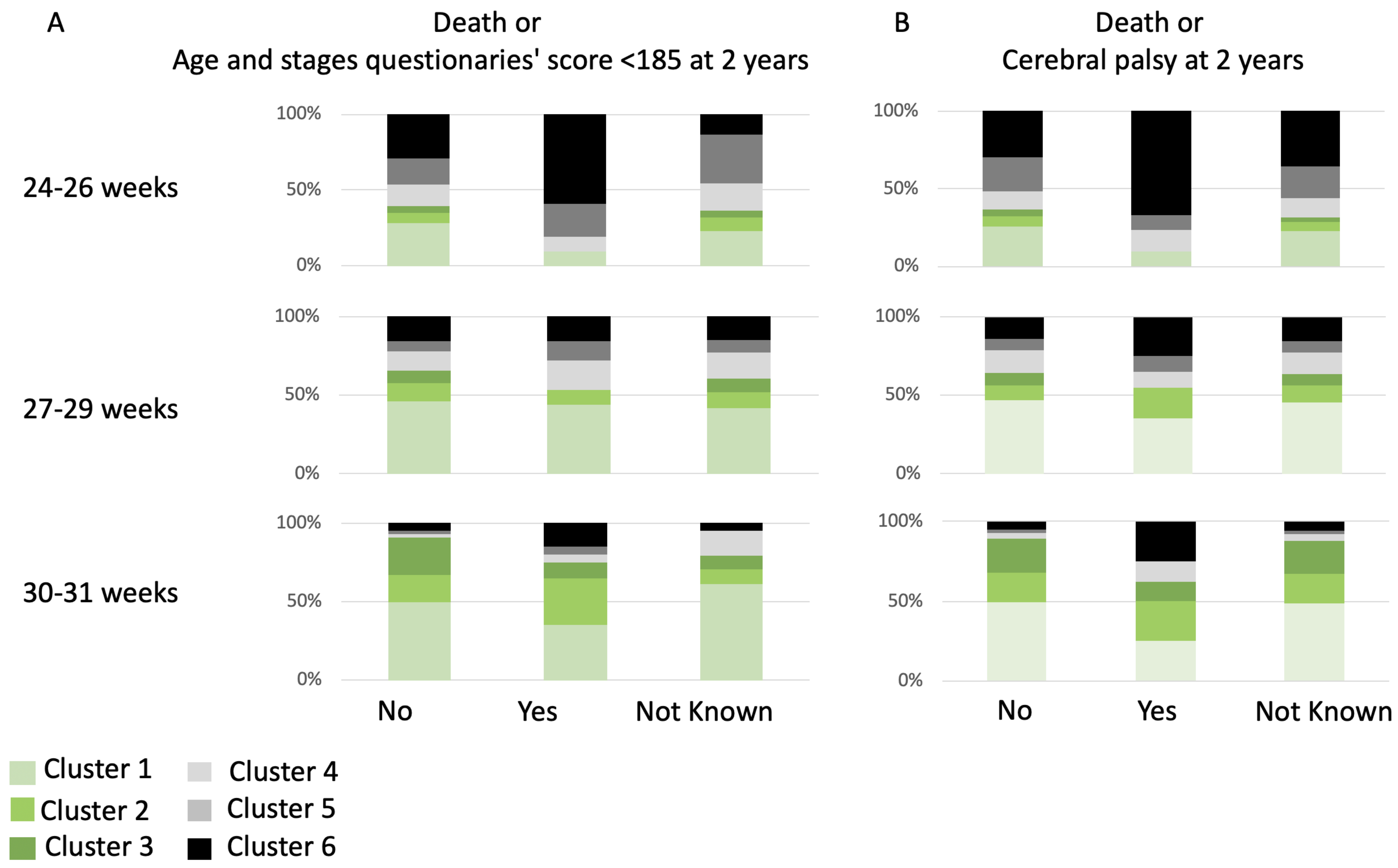

© 2020 Rozé J-C et al. JAMA Network Open.
